# Development and validation of a multivariable risk prediction model for identifying ketosis‐prone type 2 diabetes

**DOI:** 10.1111/1753-0407.13407

**Published:** 2023-05-10

**Authors:** Jia Zheng, Shiyi Shen, Hanwen Xu, Yu Zhao, Ye Hu, Yubo Xing, Yingxiang Song, Xiaohong Wu

**Affiliations:** ^1^ Geriatric Medicine Center, Key Laboratory of Endocrine Gland Diseases of Zhejiang Province, Department of Endocrinology Zhejiang Provincial People's Hospital (Affiliated People's Hospital, Hangzhou Medical College) Hangzhou People's Republic of China

**Keywords:** clinical characteristic, ketosis‐prone type 2 diabetes mellitus, nomogram, prediction model, 临床特征, 酮症倾向2型糖尿病, 列线图, 预测模型

## Abstract

**Background:**

To develop and validate a multivariable risk prediction model for ketosis‐prone type 2 diabetes mellitus (T2DM) based on clinical characteristics.

**Methods:**

A total of 964 participants newly diagnosed with T2DM were enrolled in the modeling and validation cohort. Baseline clinical data were collected and analyzed. Multivariable logistic regression analysis was performed to select independent risk factors, develop the prediction model, and construct the nomogram. The model's reliability and validity were checked using the receiver operating characteristic curve and the calibration curve.

**Results:**

A high morbidity of ketosis‐prone T2DM was observed (20.2%), who presented as lower age and fasting C‐peptide, and higher free fatty acids, glycated hemoglobin A_1c_ and urinary protein. Based on these five independent influence factors, we developed a risk prediction model for ketosis‐prone T2DM and constructed the nomogram. Areas under the curve of the modeling and validation cohorts were 0.806 (95% confidence interval [CI]: 0.760–0.851) and 0.856 (95% CI: 0.803–0.908). The calibration curves that were both internally and externally checked indicated that the projected results were reasonably close to the actual values.

**Conclusions:**

Our study provided an effective clinical risk prediction model for ketosis‐prone T2DM, which could help for precise classification and management.

## Graphical abstract

**Highlights**

- Ketosis‐prone type 2 diabetes mellitus (T2DM) is a novel type of diabetes mellitus, distinguished from type 1 diabetes mellitus and T2DM. Its clinical characteristics are complex and there is a lack of auxiliary tools for differential diagnosis.
- Ketosis‐prone T2DM is a specific but common subtype in newly diagnosed T2DM patients in China, with specific clinical characteristics.
- Based on age, fasting C‐peptide, free fatty acids, glycated hemoglobin A_1c_ and urinary protein, our study provided an effective clinical risk prediction model for ketosis‐prone T2DM, which could help for precise classification and management.

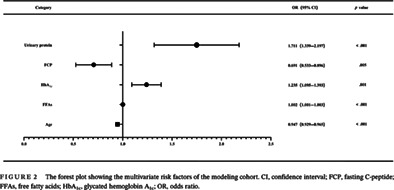

## 摘要

背景:基于临床特征构建酮症倾向2型糖尿病(T2DM)的多变量风险预测模型并进行验证。

方法:共有964名新诊断为T2DM的参与者被纳入建模和验证队列, 收集并分析基线临床资料。采用多变量logistic回归分析, 筛选独立危险因素, 建立预测模型并构建模态图。利用受试者特征曲线和标定曲线对模型的信度和效度进行检验。

结果:酮症倾向T2DM患病率较高(20.2%)。酮症倾向的T2DM患者年龄, 空腹C肽水平较低, 游离脂肪酸, 糖化血红蛋白和尿蛋白水平较高。基于这5个独立影响因素, 构建酮症倾向T2DM的风险预测模型并构建列线图。建模队列和验证队列的曲线下面积分别为0.806(95%可信区间:0.760 ~ 0.851)和0.856(95%可信区间:0.803 ~ 0.908)。内部和外部校准曲线表明, 预测结果与实际值相当接近。

结论:本研究为酮症倾向T2DM患者提供了一种有效的临床风险预测模型, 有助于对酮症倾向T2DM患者进行精准分类和管理。

## 1 INTRODUCTION

In 2019, the World Health Organization published an update on the classification of diabetes, which proposed ketosis‐prone type 2 diabetes mellitus (T2DM), distinguished from traditional type 1 diabetes mellitus and T2DM.^1^ Ketosis‐prone T2DM is a novel form of diabetes that begins with ketosis or ketoacidosis, without evident triggers. At the time of presentation, there is a temporary secretory defect of β‐cells, but it later goes into remission and does not require insulin treatment, which is significantly different from the treatment and prognosis of traditional T2DM.^2^, ^3^ Ketosis‐prone T2DM is now widely recognized in several ethnic populations, such as African‐Americans, African‐Caribbeans, and sub‐Saharan Africans.^4^, ^5^

Recently, more cases have been reported in Asian populations, including Korean, Japanese, Indian, and Chinese.^6^, ^7^, ^8^, ^9^ In China, the incidence of T2DM with ketosis onset is increasing annually.^10^ Some studies have summarized the clinical characteristics of ketosis‐prone T2DM and tried to find indicators for diabetes classification.^7^, ^11^ However, the data shown in these studies were inconsistent, the indicators did not involve complete diabetes complications and other endocrine comorbidities, and did not provide auxiliary tools for differential diagnosis.

Therefore, we aim to explore the clinical characteristics of ketosis‐prone T2DM from detailed inpatient medical records, analyze the related independent risk factors, and build a comprehensively prediction model for precise classification and management.

## 2 METHODS

### 2.1 Ethics statements

This research was carried out in conformity with the principles of the Helsinki Declaration, which was approved by the Ethics Committee of Zhejiang Provincial People's Hospital (Ethics Approval Number: 2021QT398). Informed consent was exempted, due to the retrospective nature of the data acquisition.

### 2.2 Study design and population

**FIGURE 1:**
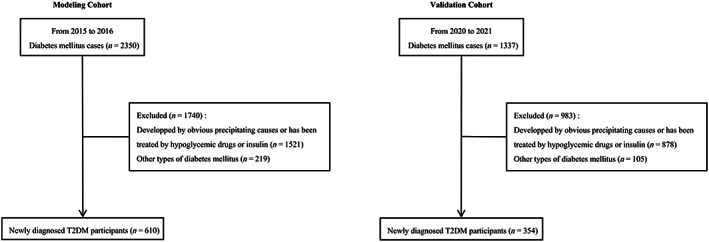
Flow chart of the enrolled patients in the modeling and validation cohorts. Participants in the modeling cohort were recruited from January 2015 to December 2016, and those in the validation cohort were recruited from January 2020 to December 2021. T2DM, type 2 diabetes mellitus.

The purpose of this cross‐sectional research was to study the clinical characteristics and develop a prediction model for ketosis‐prone T2DM. A total of 964 participants with T2DM satisfied the requirements for inclusion in the study. All participants included were from Zhejiang Provincial People's Hospital. The inclusion criteria were as follows: (a) newly diagnosed T2DM with/without ketosis (urine ketone ≥30 mg/dL with hyperglycemia) or ketoacidosis (arterial blood pH <7.30 or serum bicarbonate <18 mmol/L apart from ketosis and hyperglycemia)^1^; (b) negative for glutamate decarboxylase autoantibodies, islet cell antibodies, insulin autoantibodies, and protein tyrosine phosphatase autoantibodies; and (c) no history of treatment with hypoglycemic drugs or insulin. The exclusion criteria were as follows: (a) obvious precipitating causes for the development of ketosis, such as infection, stress, surgery, trauma, excess intake of soft drinks, and infusion of a large amount of glucose; and (b) type 1 diabetes, latent autoimmune diabetes in adults, maturity onset diabetes in the young, mitochondrial diabetes, monogenic diabetes, gestational and secondary diabetes. Participants in the modeling cohort were recruited from January 2015 to December 2016, and those in the validation cohort were recruited from January 2020 to December 2021. The workflow of participants screening is shown in Figure 1.

All participants underwent physical, laboratory, and imaging examinations and were treated with intensive insulin therapy. The participants with ketosis or ketoacidosis were included in the ketosis‐prone T2DM group.

### 2.3 Data collection

We collected detailed information on the participants' general characteristics, such as gender, age, smoking, drinking, weight loss in the last 6 months, the onset pattern of diabetes, family history of diabetes, diabetes duration, everyday risk factors, past health records, and medication histories. Participant's height, weight, systolic blood pressure, and diastolic blood pressure were measured by the same nurse on the first day of admission. To determine body mass index, divide weight by square of height.

We collected blood and urine samples in the morning, following an 8‐hour fast by the participant. Alanine aminotransferase (ALT), aspartate aminotransferase, blood urea nitrogen (BUN), creatinine, uric acid, triglyceride, total cholesterol, high‐density lipoprotein cholesterol (HDL‐C), low‐density lipoprotein cholesterol, free fatty acids (FFAs), glycated hemoglobin A_1c_ (HbA_1c_), microalbumin‐to‐creatinine ratio, β‐C terminal cross‐linking telopeptide of type I collagen (β‐CTX), *N*‐terminal propeptide of type I procollagen, parathyroid hormone, 25‐hydroxy vitamin D, fasting plasma glucose (FPG), 2‐hour postprandial plasma glucose (2‐h‐PPG), fasting serum insulin (FINS), 2‐hour postprandial serum insulin (2‐h‐PINS), fasting C‐peptide (FCP), 2‐hour postprandial C‐peptide (2‐h‐PCP), urinary ketosis, and urinary protein were measured by chemiluminescence immunoassay or standardized high‐performance liquid chromatography assay. Insulin or its analogs were administered to participants with newly diagnosed T2DM after ketosis or ketoacidosis had been treated. The FPG level <7.0 mmol/L and 2‐h‐PPG level < 11.1 mmol/L were used as the blood glucose control target. After blood glucose control reached the standard, venous blood was collected to measure FPG, 2‐h‐PPG, FINS, 2‐h‐PINS, FCP, and 2‐h‐PCP levels the next morning. Free triiodothyronine (FT3), free thyroxine, and thyroid‐stimulating hormone were measured by chemiluminescent immunoassay. The assessments were all conducted in the same laboratory. The following equations were used to determine the homeostasis model assessment of insulin resistance (HOMA‐IR) and the homeostasis model assessment of β‐cell function (HOMA‐β): HOMA‐IR = fasting serum insulin × FPG/22.5, HOMA‐β = 20 × fasting serum insulin/(FPG – 3.5).

Lower‐limb atherosclerosis and carotid atherosclerosis were evaluated using a high‐end color Doppler ultrasound scanner (EPIQ7, Philips). Cerebral infarction was analyzed using 3.0 T magnetic resonance imaging scanner (Discovery MR 750, GE). Peripheral neuropathy was evaluated using six‐channel electromyography/evoked potential (keypoint).

### 2.4 Definitions

A regimen of intensive insulin therapy comprised the use of an external insulin infusion pump or at least three daily insulin injections. Time for FPG to reach target was defined from the date of admission to the day of returning to normoglycemia or discharge. The proportion of 10‐year high atherosclerotic cardiovascular disease (ASCVD) risk was evaluated using ASCVD risk assessments from the 2020 Chinese Guideline on the Primary Prevention of Cardiovascular Diseases.^12^

### 2.5 Statistical analysis

The Kolmogorov–Smirnov test was used to determine the index's distribution. Means and SDs were used to represent normally distributed data, and nonnormally distributed data were descriptive by medians and quartiles. Counts and proportions were used to describe categorical variables. To distinguish any differences between the two groups, the Student *t* test or the nonparametric Mann–Whitney *U* test were used. The discrepancies in categorical variables were compared through the *χ* ^2^ test.

Stepwise regression analysis was used to calculate *p* values for trend (*p*‐trend) to determine the independent association between the incidence of ketosis‐prone T2DM and different HbA_1c_ levels. Model 1 was gender and age adjusted. Model 2 was additionally modified for FFAs and FCP. Univariate logistic regression analyses were used to identify independent risk factors, and multivariate analysis was carried out using logistic regression analysis with variables that were meaningful in univariate testing included (*p* < .05).

Forest plots of hazard ratios were plotted based on the aforementioned data. Using the nomogram function from the rms library, we created a nomogram to predict ketosis‐prone T2DM. The score's accuracy was determined by calculating the receiver operating characteristic curve and analyzing the area under the receiver operating characteristic curve (AUC). The modeling cohort was compared to the validation cohort by assessing the concordance index and calibration curves on the test set to verify the performance of the model. SPSS, version 25.0 (IBM Corp.) and R, version 3.6 were used for all analyses. Differences were considered statistically significant at *p* < .05.

## 3 RESULTS

### 3.1 Baseline characteristics of the study population

In total, 964 participants were included in the analysis, with 610 participants in the modeling cohort and 354 participants in the validation cohort. The incidence rate of ketosis‐prone T2DM was 21.0% (128 participants) and 18.9% (67 participants), respectively. According to the *χ* ^2^ analysis, the incidence rate was not significantly different (*p* > .05). Baseline characteristics of participants enrolled in both studies were broadly similar. (Table 1).

**TABLE 1:** Clinical characteristics of the modeling and validation cohorts.

	Modeling cohort (*n* = 610)	Validation cohort (*n* = 354)
Men (%)	436 (71.5%)	276 (78.0%)
Age (years)	49.00 (39.00, 58.00)	48.00 (37.00, 57.00)
SBP (mmHg)	135.00 (124.00, 145.00)	127.00 (116.00, 140.00)
DBP (mmHg)	84.00 (75.00, 91.00)	80.00 (72.00, 86.50)
BMI (kg/m^2^)	24.48 (22.48, 27.27)	25.16 (22.88, 27.75)
Current smoker (%)	206 (33.8%)	136 (39.0%)
Current drinker (%)	159 (26.1%)	150 (43.1%)
Family history of diabetes (%)	163 (26.7%)	116 (33.2%)
ALT (U/L)	26.00 (17.00, 43.00)	30.00 (19.00, 51.00)
AST (U/L)	23.00 (18.00, 31.00)	25.00 (19.00, 39.00)
BUN (mmol/L)	4.86 (4.02, 5.73)	4.73 (3.84, 5.61)
Cr (μmol/L)	77.85 (69.50, 87.00)	75.50 (64.60, 83.20)
UA (μmol/L)	295.00 (242.00, 357.00)	310.00 (260.50, 379.50)
TC (mmol/L)	5.00 (4.38, 5.78)	4.95 (4.25, 5.65)
TG (mmol/L)	1.69 (1.15, 2.88)	1.51 (1.01, 2.21)
HDL‐C (mmol/L)	0.98 (0.83, 1.15)	0.97 (0.84, 1.14)
LDL‐C (mmol/L)	2.98 (2.39, 3.60)	2.95 (2.42, 3.50)
FFAs (μmol/L)	558.00 (390.75, 750.25)	472.00 (338.00, 617.00)
HbA_1c_ (mmol/mol)	98 ± 19	92 ± 19
HbA_1c_ (%)	11.1 ± 2.1	10.6 ± 2.1
FPG (mmol/L)	6.58 (5.24, 9.66)	6.69 (5.30, 10.10)
2‐h‐PPG (mmol/L)	11.60 (9.06, 15.89)	16.17 (13.76, 18.62)
FINS (μIU/mL)	12.67 (8.80, 16.92)	4.15 (1.43, 9.13)
2‐h‐PINS (μIU/mL)	48.26 (31.46, 66.17)	25.64 (14.51, 46.32)
FCP (ng/mL)	1.51 (0.95, 2.27)	1.25 (0.73, 2.06)
2‐h‐PCP (ng/mL)	3.65 (2.27, 5.23)	5.23 (3.61, 7.36)
HOMA‐IR	3.78 (2.55, 5.55)	1.40 (0.38, 3.54)
HOMA‐β	82.44 (35.70, 154.59)	24.14 (9.30, 57.55)
Urinary protein (%)		
‐	314 (51.5%)	193 (54.5%)
± ~ +	264 (43.3%)	144 (40.7%)
++ ~ ++++	32 (5.2%)	17 (4.8%)
Ketosis‐prone T2DM (%)	128 (21.0%)	67 (18.9%)

*Note*: Results are presented as *N* (%) or mean ± SD or median (quartile 1 − quartile 3).

Abbreviations: 2‐h‐PCP, 2‐hour postprandial C‐peptide; 2‐h‐PINS, 2‐hour postprandial serum insulin; 2‐h‐PPG, 2‐hour postprandial plasma glucose; ALT, alanine aminotransferase; AST, aspartate aminotransferase; BMI, body mass index; BUN, blood urea nitrogen; Cr, creatinine; DBP, diastolic blood pressure; FCP, fasting C‐peptide; FFAs, free fatty acids; FINS, fasting serum insulin; FPG, fasting plasma glucose; HbA_1c_, glycated hemoglobin A_1c_; HDL‐C, high‐density lipoprotein cholesterol; HOMA‐IR, homeostasis model assessment of insulin resistance; HOMA‐β, homeostasis model assessment of β‐cell function; LDL‐C, low‐density lipoprotein cholesterol; SBP, systolic blood pressure; TC, total cholesterol; T2DM, type 2 diabetes mellitus; TG, triglyceride; UA, uric acid.

The clinical characteristics of ketosis‐prone T2DM are shown in Table 2. Ketoacidosis accounted for 23.4% of ketosis‐prone T2DM. Obviously, participants with ketosis‐prone T2DM were more likely to discovered because of the symptom such as thirst, polyuria etc. at the onset pattern of diabetes and presented with more weight loss (all, *p* < .001). These participants were markedly younger (42.50, interquartile range [IQR], 33.00–52.00), had higher levels of FFAs, HbA_1c_, urinary protein, longer duration of intensive insulin therapy, and time for FPG to reach target, whereas those levels of HDL‐C, FCP, 2‐h‐PCP, ALT, and BUN were significantly reduced (all, *p* < .05). They were more likely to require insulin therapy at discharge (*p* < .001). Other clinical data were not significantly different between the two groups (all, *p* > .05).

**TABLE 2:** Clinical characteristics of ketosis‐prone T2DM and non‐ketotic T2DM.

	Ketosis‐prone T2DM (*n* = 128)	Non‐ketotic T2DM (*n* = 482)	*p* value
Men (%)	96 (75.0%)	340 (70.5%)	.320
Age (years)	42.50 (33.00, 52.00)	51.00 (42.00, 60.00)	<.001
SBP (mmHg)	133.00 (122.00, 141.00)	134.00 (123.00, 147.00)	.094
DBP (mmHg)	82.77 ± 11.84	82.40 ± 10.78	.737
BMI (kg/m^2^)	23.96 (21.86, 27.46)	24.57 (22.58, 27.11)	.364
Weight loss (kg)	5.00 (0.00, 7.50)	2.25 (0.00, 5.00)	<.001
Current smoker (%)	41 (32.0%)	165 (34.2%)	.640
Current drinker (%)	29 (22.7%)	130 (27.0%)	.323
The onset pattern of diabetes (%)			<.001
Physical examinations	10 (7.8%)	122 (25.3%)	
Symptoms	118 (92.2%)	360 (74.7%)	
Family history of diabetes (%)	34 (26.6%)	129 (26.8%)	.964
ALT (U/L)	22.00 (14.75, 38.25)	26.00 (17.00, 45.00)	.037
AST (U/L)	22.00 (17.00, 30.00)	23.00 (18.00, 32.00)	.115
BUN (mmol/L)	4.51 (3.22, 5.53)	4.99 (4.16, 5.80)	<.001
Cr (μmol/L)	76.80 (68.13, 88.43)	77.60 (69.20, 86.70)	.697
UA (μmol/L)	300.00 (234.75, 375.75)	292.00 (242.00, 350.00)	.336
TC (mmol/L)	5.02 (4.27, 6.33)	5.00 (4.35, 5.68)	.141
TG (mmol/L)	1.69 (1.09, 3.16)	1.73 (1.16, 2.80)	.855
HDL‐C (mmol/L)	0.90 (0.74, 1.18)	0.99 (0.86, 1.16)	.004
LDL‐C (mmol/L)	2.84 (2.30, 3.76)	2.99 (2.41, 3.57)	.589
FFAs (μmol/L)	643.00 (497.00, 892.00)	504.50 (362.00, 697.00)	<.001
HbA_1c_ (mmol/mol)	107 ± 18	94 ± 19	<.001
HbA_1c_ (%)	11.9 ± 2.0	10.8 ± 2.1	<.001
FPG (mmol/L)	6.54 (5.54, 9.23)	6.61 (5.26, 9.68)	.601
2‐h‐PPG (mmol/L)	11.50 (8.37, 15.41)	11.65 (9.17, 15.89)	.268
FINS (μIU/mL)	12.95 (9.10, 16.68)	12.71 (8.74, 17.14)	.758
2‐h‐PINS (μIU/mL)	44.55 (29.90, 60.15)	49.80 (31.46, 68.66)	.111
FCP (ng/mL)	1.21 (0.76, 1.97)	1.57 (0.99, 2.38)	.001
2‐h‐PCP (ng/mL)	2.81 (1.44, 4.11)	3.79 (2.36, 5.45)	<.001
HOMA‐IR	3.97 (2.48, 5.31)	3.72 (2.55, 5.60)	.793
HOMA‐β	81.52 (31.48, 125.31)	72.56 (28.71, 150.29)	.729
Urinary protein (%)			<.001
‐	36 (28.1%)	278 (57.7%)	
± ~ +	82 (64.1%)	182 (37.8%)	
++ ~ ++++	10 (7.8%)	22 (4.6%)	
Duration of intensive insulin therapy (days)	9.00 (7.00, 11.00)	8.00 (7.00, 10.00)	.015
Time for fasting plasma glucose to reach target (days)	4.00 (3.00, 6.00)	3.00 (2.00, 5.00)	<.001
Discharge treatment (%)			<.001
Diet and exercise control	0 (0.0%)	14 (2.9%)	
Oral hypoglycemic agents	77 (60.2%)	350 (72.6%)	
Premixed insulin with/without oral hypoglycemic agents	47 (36.7%)	113 (23.4%)	
Basal and mealtime insulin	4 (3.1%)	5 (1.0%)	

*Note*: *p* value of the Student *t* test, nonparametric Mann–Whitney *U* test or *χ* ^2^ test in comparison between ketosis‐prone T2DM and nonketotic T2DM groups. Results are presented as *N* (%) or mean ± SD or median (quartile 1 − quartile 3).

Abbreviations: 2‐h‐PCP, 2‐hour postprandial C‐peptide; 2‐h‐PINS, 2‐hour postprandial serum insulin; 2‐h‐PPG, 2‐hour postprandial plasma glucose; ALT, alanine aminotransferase; AST, aspartate aminotransferase; BMI, body mass index; BUN, blood urea nitrogen; Cr, creatinine; DBP, diastolic blood pressure; FCP, fasting C‐peptide; FFAs, free fatty acids; FINS, fasting serum insulin; FPG, fasting plasma glucose; HbA_1c_, glycated hemoglobin A_1c_; HDL‐C, high‐density lipoprotein cholesterol; HOMA‐IR, homeostasis model assessment of insulin resistance; HOMA‐β, homeostasis model assessment of β‐cell function; LDL‐C, low‐density lipoprotein cholesterol; SBP, systolic blood pressure; TC, total cholesterol; T2DM, type 2 diabetes mellitus; TG, triglyceride; UA, uric acid.

### 3.2 Associations of different levels of HbA_1c_ with ketosis‐prone T2DM

The associations between different HbA_1c_ levels and ketosis‐prone T2DM with multistep adjustments are shown in Table 3. The likelihood of ketosis‐prone T2DM tended to be elevated with an increase in the HbA_1c_ level (*p*‐trend<.001). The results presented herein indicated that the increasing trend persisted even after correction for gender and age (*p*‐trend<.001). When further adjusting for FFAs and FCP, the growing trend remained independently associated with HbA_1c_ levels (*p*‐trend = .001).

**TABLE 3:** The OR and 95% CI of different HbA_1c_ levels with ketosis‐prone T2DM.

Variable	Univariate			Model 1			Model 2		
	OR	95% CI	*p* value	OR	95% CI	*p*‐value	OR	95% CI	*p* value
HbA_1c_, %									
<9.6	1			1			1		
9.6 ≤ HbA_1c_ < 10.9	2.110	1.021–4.362	.044	1.937	0.926–4.051	.079	2.002	0.900–4.451	.089
10.9 ≤ HbA_1c_ < 12.4	3.421	1.740–6.727	<.001	3.344	1.681–6.654	.001	3.385	1.570–7.297	.002
HbA_1c_ ≥ 12.4	4.466	2.291–8.708	<.001	3.980	2.012–7.873	<.001	3.125	1.449–6.740	.004
*p*‐trend	<.001			<.001			.001		

*Note*: Model 1 is adjusted for gender and age. Model 2 is adjusted for gender, age, FFAs, and FCP.

Abbreviations: CI: confidence interval; FCP, fasting C‐peptide; FFAs, free fatty acids; HbA_1c_, glycated hemoglobin A_1c_; OR: odds ratio; T2DM, type 2 diabetes mellitus.

### 3.3 Ketosis‐prone T2DM with diabetes complications and endocrine comorbidities

A comparison of diabetes complications is shown in Table 4. Evidently, the incidence of diabetes complications in the ketosis‐prone T2DM group was much lower, regardless of vascular disease or nephropathy. There were obvious differences in lower limb atherosclerosis, carotid atherosclerosis, and the proportion of 10‐year high ASCVD risk (all, *p* < .05). At the same time, the differences in thyroid and bone metabolism are also described (Table 4). Levels of β‐CTX was significantly higher in the ketosis‐prone T2DM group (*p* = .010). In contrast, FT3 levels were considerably higher in the nonketotic T2DM group (*p* = .001). Furthermore, other variable distributions did not correlate between the two groups (all, *p* > .05).

**TABLE 4:** Comparison of diabetes complications and endocrine comorbidities in two groups.

	Ketosis‐prone T2DM	Non‐ketotic T2DM	*p* value
Comparison of diabetes complications			
Lower limb atherosclerosis	42/77 (35.3%)	223/240 (48.2%)	.012
Carotid atherosclerosis	37/84 (30.6%)	220/240 (47.8%)	.001
Cerebral infarction	12/34 (26.1%)	104/153 (40.5%)	.065
Proportion of 10‐year high ASCVD risk	81/47 (63.3%)	392/90 (81.3%)	<.001
ACR (mg/g)	14.14 (8.66, 35.63)	15.03 (7.96, 34.48)	.786
Peripheral neuropathy	18/30 (37.5%)	92/86 (51.7%)	.081
Comparison of endocrine comorbidities			
TSH (mIU/L)	1.43 (0.97, 2.26)	1.49 (0.99, 2.21)	.636
FT3 (ng/L)	2.85 (2.51, 3.13)	2.98 (2.68, 3.31)	.001
FT4 (ng/L)	10.24 (9.40, 11.20)	9.94 (8.94, 11.17)	.069
β‐CTX (pg/ml)	546.00 (382.50, 771.50)	409.00 (271.50, 600.00)	.010
P1NP (ng/mL)	37.63 (23.59, 54.04)	38.13 (27.58, 47.68)	.907
PTH (pg/mL)	39.55 (26.10, 47.98)	39.50 (31.60, 51.10)	.395
25‐OHD (ng/mL)	13.90 (10.93, 16.05)	15.60 (11.53, 19.66)	.080

*Note*: Results are presented as median (quartile 1 − quartile 3) or positive/negative number (positive percent).

Abbreviations: 25‐OHD, 25‐hydroxy vitamin D; ACR, microalbumin‐to‐creatinine ratio; ASCVD, atherosclerotic cardiovascular disease; β‐CTX, β‐C terminal cross‐linking telopeptide of type I collagen; FT3, free triiodothyronine; FT4, free thyroxine; P1NP, N‐terminal propeptide of type I procollagen; PTH, parathyroid hormone; T2DM: type 2 diabetes mellitus; TSH, thyroid‐stimulating hormone.

### 3.4 Findings of univariate and multivariate logistic regression analysis

**FIGURE 2:**
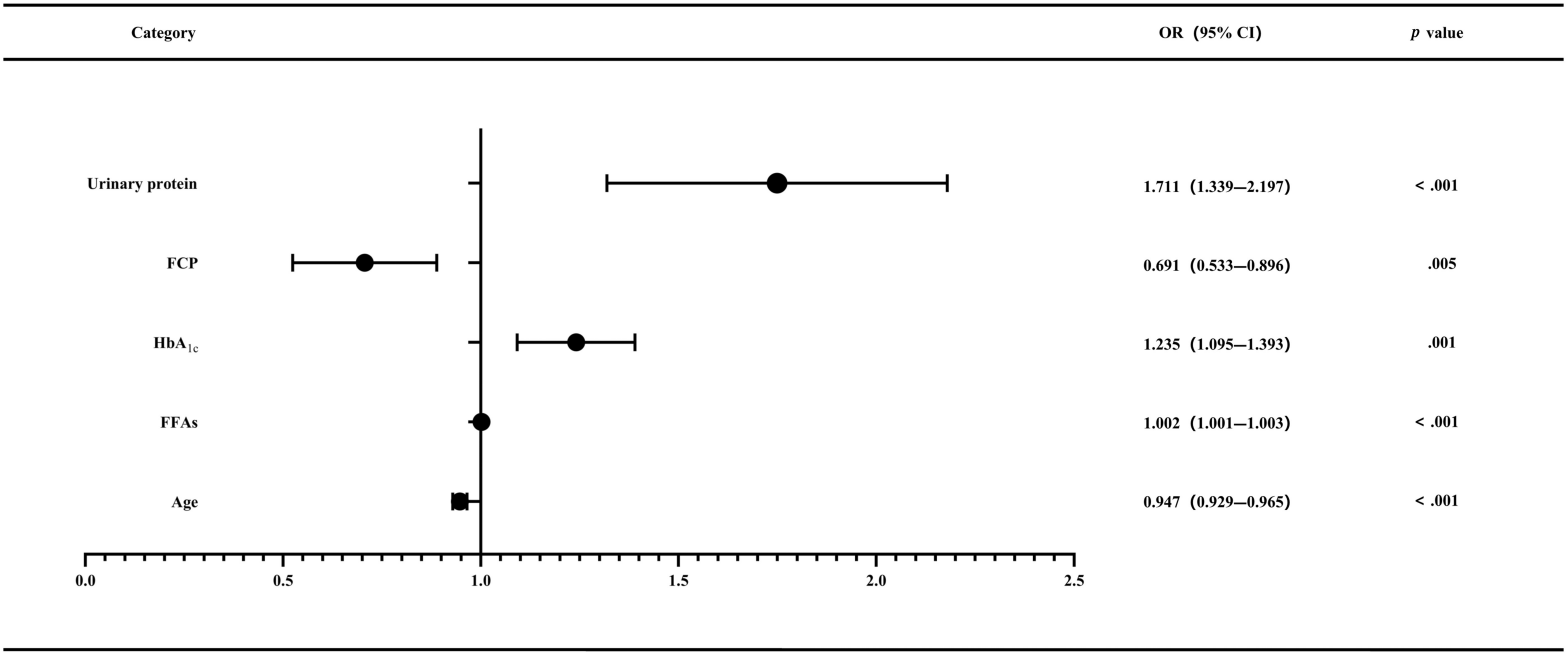
The forest plot showing the multivariate risk factors of the modeling cohort. CI, confidence interval; FCP, fasting C‐peptide; FFAs, free fatty acids; HbA_1c_, glycated hemoglobin A_1c_; OR, odds ratio.

Univariate logistic regression analysis demonstrated that factors such as age, HDL‐C, FFAs, HbA_1c_, FCP, 2‐h‐PCP, urinary protein, FT3, β‐CTX, lower limb atherosclerosis, carotid atherosclerosis, proportion of 10‐year high ASCVD risk, duration of intensive insulin therapy, and time for FPG to reach target were statistically significant (all, *p* < .05). All the listed variables were included in the multivariate logistic regression analysis. The results revealed that FFAs (odds ratio [OR] = 1.002, 95% confidence interval [CI]: 1.001–1.003, *p* < .001), HbA_1c_ (OR = 1.235, 95% CI: 1.095–1.393, *p* = .001), and urinary protein (OR = 1.711, 95% CI: 1.339–2.197, *p* < .001) were independent risk factors, whereas age (OR = 0.947, 95% CI: 0.929–0.965, *p* < .001) and FCP (OR = 0.691, 95% CI: 0.533–0.896, *p* = .005) were independent protective factors for the incidence of ketosis‐prone T2DM (Table 5, Figure 2). A model for predicting the incidence of ketosis‐prone T2DM was constructed based on the aforementioned results.

**TABLE 5:** Factors are selected for predicting ketosis‐prone T2DM by logistic regression analysis.

	Univariate analysis			Multivariate analysis		
	OR	95% CI	*p* value	OR	95% CI	*p* value
Age (years)	0.955	0.940–0.971	<.001	0.947	0.929–0.965	<.001
ALT (U/L)	0.998	0.992–1.003	.379			
BUN (mmol/L)	0.885	0.774–1.011	.072			
HDL‐C (mmol/L)	0.441	0.199–0.977	.044			
FFAs (μmol/L)	1.002	1.001–1.003	<.001	1.002	1.001–1.003	<.001
HbA_1c_ (%)	1.288	1.168–1.420	<.001	1.235	1.095–1.393	.001
FCP (ng/mL)	0.706	0.565–0.883	.002	0.691	0.533–0.896	.005
2‐h‐PCP (ng/mL)	0.769	0.689–0.857	<.001			
Urinary protein (%)	1.759	1.438–2.151	<.001	1.711	1.339–2.197	<.001
FT3 (ng/L)	0.408	0.249–0.669	<.001			
β‐CTX (pg/ml)	1.002	1.000–1.004	.014			
Lower limb atherosclerosis	0.587	0.387–0.892	.012			
Carotid atherosclerosis	0.481	0.313–0.737	.001			
Proportion of 10‐year high ASCVD risk	0.400	0.260–0.614	<.001			
Duration of intensive insulin therapy (days)	1.073	1.002–1.148	.042			
Time for fasting plasma glucose to reach target (days)	1.159	1.074–1.252	<.001			

Abbreviations: 2‐h‐PCP, 2‐hour postprandial C‐peptide; ALT, alanine aminotransferase; ASCVD, atherosclerotic cardiovascular disease; BUN, blood urea nitrogen; β‐CTX, β‐C terminal cross‐linking telopeptide of type I collagen; CI, confidence interval; FCP, fasting C‐peptide; FFAs, free fatty acids; FT3, free triiodothyronine; HbA_1c_, glycated hemoglobin A_1c_; HDL‐C, high‐density lipoprotein cholesterol; OR, odds ratio; T2DM, type 2 diabetes mellitus.

### 3.5 Construction and evaluation of the prediction model

**FIGURE 3:**
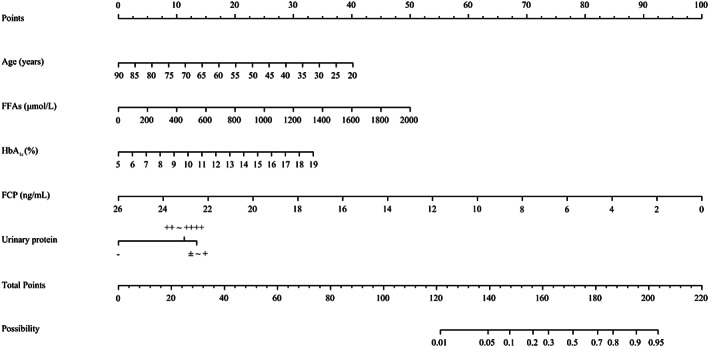
Nomogram for predicting ketosis‐prone T2DM. The probability of ketosis‐prone T2DM was determined by calculating the corresponding score for each risk factor. FCP, fasting C‐peptide; FFAs, free fatty acids; HbA_1c_, glycated hemoglobin A_1c_; T2DM, type 2 diabetes mellitus.

**FIGURE 4:**
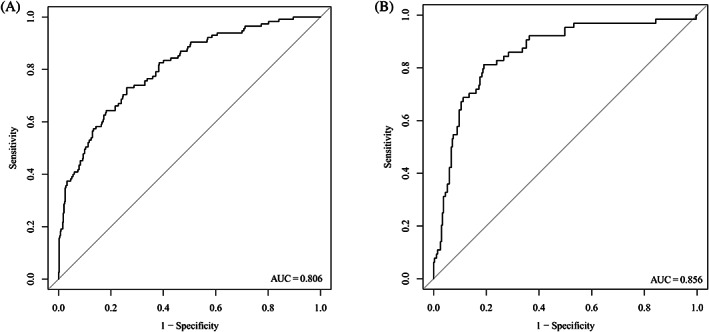
The ROC curves of the model in the modeling and validation cohorts. (A) modeling cohort, (B) validation cohort. The AUC of the model in the modeling cohort was 0.806 (95% CI: 0.760–0.851), whereas it was 0.856 (95% CI: 0.803–0.908) in the validation cohort. AUC, area under the receiver operating characteristic curve; CI, confidence interval; ROC, receiver operating characteristic.

**FIGURE 5:**
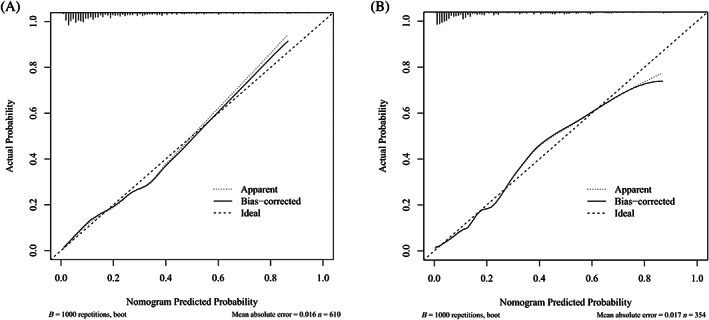
The calibration curves of the prediction model for ketosis‐prone T2DM. (A) modeling cohort, (B) validation cohort. The calibration curves of modeling cohort and validation cohort both are close to a straight line with a slope of 1. T2DM, type 2 diabetes mellitus.

We created a nomogram to predict ketosis‐prone T2DM (Figure 3). The probability of ketosis‐prone T2DM was determined by calculating the corresponding score for each risk factor. As shown in Figure 4, the AUC of the model in the modeling cohort was 0.806 (95% CI: 0.760–0.851), whereas it was 0.856 (95% CI: 0.803–0.908) in the validation cohort. The calibration curves of the model in the modeling and validation cohorts are shown in Figure 5. The projected findings were close to the actual values, according to both internally and externally confirmed calibration curves.

## 4 DISCUSSION

This study revealed that ketosis‐prone T2DM was common (20.2%) in newly diagnosed T2DM, characterized by younger age, poorer islet function, severe hyperglycemia and dyslipidemia, higher levels of FFAs, HbA_1c_, urinary protein, longer duration of intensive insulin therapy and time for FPG to reach target, and lower proportion of macrovascular disease. Based on independent influence factors such as age, FCP, FFAs, HbA_1c_, and urinary protein, we developed the first predictive model for ketosis‐prone T2DM, with an excellent discriminatory power of 0.806 while maintaining adequate calibration.

Since the 1980s, it has been recognized that some participants with ketoacidosis may have a clinical course more like that of T2DM than type 1 diabetes mellitus, which was called ketosis‐prone T2DM.^2^, ^13^ Recently, with the increasing awareness of ketosis‐prone T2DM, the morbidity has gradually increased.^14^ Zhang et al reported that ketosis‐prone T2DM participants accounted for 7.6% of the participants with diabetes requiring admission to a large urban hospital in China.^15^ Lontchi‐Yimagou et al found 28.3% ketosis‐prone T2DM of the hyperglycemic crises.^16^ We observed 20.2% ketosis‐prone T2DM in newly diagnosed T2DM in our center. The discrepancy might be caused by the various types of participants recruited. As a Grade‐A tertiary referral hospital in local region, our center will admit more participants with severe conditions.

In our study, we explored the clinical characteristics of participants at the onset of ketosis‐prone T2DM. First, ketosis‐prone T2DM participants were diagnosed at lower age and predominantly middle‐aged men, which was consistent with previous studies.^2^, ^8^ Genetic factors, changes in hormone levels, and environmental factors might all interact to cause the condition. Second, ketosis‐prone T2DM had a temporary secretory defect of β‐cells. Mauvais‐Jarvis et al^17^ suggested that the ketosis‐prone T2DM included one major subtype with preserved insulin secretion and a rarer subgroup with permanent insulin dependence. Our participants showed lower levels of FCP at the onset. Third, glucose metabolic disturbance was more severe in ketosis‐prone T2DM participants, which was in line with previous studies.^3^, ^8^ Our study provided further evidence of the relationship between HbA_1c_ levels and the development of ketosis‐prone T2DM. Fourth, dyslipidemia was evident in ketosis‐prone T2DM participants. The role of lipotoxicity in the β‐cell failure in ketosis‐prone T2DM participants was debatable. Some studies have suggested that lipotoxicity inhibited insulin gene expression^18^ whereas Umpierrez et al discovered that intralipid infusion for 48 h was not affiliated with β‐cell decompensation in ketosis‐prone T2DM participants.^19^ Other teams used metabolomic approaches to demonstrate that ketosis was due to decreased ketolysis rather than overproduction of lipid.^20^, ^21^ In our study, a markedly higher level of FFAs and lower HDL‐C levels were seen. There was no significant difference in other lipid parameters. Fifth, ketosis‐prone T2DM had a significantly higher rate of abnormally elevated proteinuria, which was consistent with the findings of Zuo et al.^22^ It has been shown that β‐hydroxybutyric acid can cause a significant increase in waveform protein expression and a significant decrease in E‐calmodulin levels or cause some degree of kidney damage by regulating the epithelial‐mesenchymal transition of renal tubular epithelial cells via the transforming growth factor‐beta/Smad3 signaling pathway.^23^, ^24^, ^25^

In our study, we further explored diabetes complications and endocrine comorbidities in ketosis‐prone T2DM participants. First, ketosis‐prone T2DM participants had a lower rate of combined macrovascular disease at the time of onset. This might be that participants with ketosis‐prone T2DM see their doctor earlier than those without due to ketosis symptoms. However, Li's team considered that the prevalence of carotid atherosclerosis or lower limb atherosclerosis in the ketosis‐prone T2DM resembled those in the nonketotic T2DM^26^, ^27^ whereas Wang et al^11^ discovered that the prevalence of atherosclerosis was higher in the ketosis‐prone T2DM group. This disparity may be due to the different study population. Second, ketosis‐prone type 2 diabetes participants showed more severe bone destruction and more pronounced thyroid dysfunction. A similar conclusion was reached by Xu et al that ketosis or ketoacidosis may reduce the activity of osteoblasts, increase the activity of osteoclasts, and lead to β‐CTX increase, while damaging the hypothalamic–pituitary‐thyroid axis, resulting in low T3 syndrome.^28^

### 4.1 Limitations

Several limitations in the current study should be addressed. First, because our center is a regional tertiary referral hospital with admissions of participants with relatively severe conditions, there may be a selection bias in the study population. But the homogeneous treatment process can help to reflect the characteristics of ketosis‐prone T2DM at the time of onset. Second, our study can only show the correlations between the risks and ketosis‐prone T2DM; it cannot explain causality. The underlying mechanisms need to be investigated. Third, a single cross‐sectional design only reflects the characteristics of ketosis‐prone T2DM participants at the time of onset and the long‐term outcomes need further exploration. Fourth, the modeling and validation cohorts come from the same center, which implies that the results need to be further evaluated in a larger population.

### 4.2 Future directions

Our study comprehensively showed the clinical characteristics of ketosis‐prone T2DM and suggested the importance and necessity of identifying and managing this particular subtype. However, some related issues still need further clarification. What are the predisposing factors that contribute to the development of ketosis‐prone T2DM? How about the pathogenesis? Which drugs are more suitable for the management of ketosis‐prone T2DM? Are the long‐term outcomes of ketosis‐prone T2DM distinctive from other types of T2DM, such as islet function, complications, etc.? Therefore, longitudinal prospective cohort studies will be needed to explore these questions.

## 5 CONCLUSION

In summary, ketosis‐prone T2DM is a specific but common subtype in newly diagnosed T2DM participants in China, with specific clinical characteristics. Based on age, FCP, FFAs, HbA_1c_, and urinary protein, our study provided an effective clinical risk prediction model for ketosis‐prone T2DM, with an excellent discriminatory power of 0.806 while maintaining adequate calibration, which could help for precise classification and management.

## AUTHOR CONTRIBUTIONS

Jia Zheng analyzed the data and wrote the paper. Yu Zhao and Xiaohong Wu contributed to study conceptualization. Shiyi Shen and Hanwen Xu researched the data. Ye Hu, Yubo Xing, and Yingxiang Song conducted data quality check. All the authors have approved the final version of article and were involved in the decision to submit the manuscript for publication.

## FUNDING INFORMATION

This work was supported by the National Natural Science Foundation of China (81970714), Science and technology innovation leading talent project of Zhejiang 10 000 people plan (2021R52022) and Zhejiang province health innovative talents project (2021‐CXRC07‐01).

## CONFLICT OF INTEREST STATEMENT

The authors declare that they have no known competing financial interests or personal relationships that could have appeared to influence the work reported in this paper.

## Data Availability

The data from this study are available from the corresponding author upon reasonable request. The authors declare that they have no conflict of interest.
